# Hydrocortisone Promotes Differentiation of Mouse Embryonic Stem Cell-Derived Definitive Endoderm toward Lung Alveolar Epithelial Cells

**DOI:** 10.22074/cellj.2019.5521

**Published:** 2018-08-07

**Authors:** Mohammad Reza Mokhber Dezfouli, Sirous Sadeghian Chaleshtori, Azadeh Moradmand, Mohsen Basiri, Hossein Baharvand, Yaser Tahamtani

**Affiliations:** 1 Department of Internal Medicine, Faculty of Veterinary Medicine, University of Tehran, Tehran, Iran; 2 Institute of Biomedical Research, Faculty of Veterinary Medicine, University of Tehran, Tehran, Iran; 3 Department of Stem Cells and Developmental Biology, Cell Science Research Center, Royan Institute for Stem Cell Biology and Technology, ACECR, Tehran, Iran; 4 Department of Developmental Biology, University of Science and Culture, ACECR, Tehran, Iran

**Keywords:** Differentiation, Embryonic Stem Cells, Lung, Regenerative Medicine

## Abstract

**Objective:**

The ability to generate lung alveolar epithelial type II (ATII) cells from pluripotent stem cells (PSCs) enables the study of lung development, regenerative medicine, and modeling of lung diseases. The establishment of defined, scalable differentiation methods is a step toward this goal. This study intends to investigate the competency of small molecule induced mouse embryonic stem cell-derived definitive endoderm (mESC-DE) cells towards ATII cells.

**Materials and Methods:**

In this experimental study, we designed a two-step differentiation protocol. mESC line Royan B20 (RB20) was induced to differentiate into DE (6 days) and then into ATII cells (9 days) by using an adherent culture method. To induce differentiation, we treated the mESCs for 6 days in serum-free differentiation (SFD) media and induced them with 200 nM small molecule inducer of definitive endoderm 2 (IDE2). For days 7-15 (9 days) of induction, we treated the resultant DE cells with new differentiation media comprised of 100 ng/ml fibroblast growth factor (FGF2) (group F), 0.5 μg/ml hydrocortisone (group H), and A549 conditioned medium (A549 CM) (group CM) in SFD media. Seven different combinations of factors were tested to assess the efficiencies of these factors to promote differentiation. The expressions of DE- and ATII-specific markers were investigated during each differentiation step.

**Results:**

Although both F and H (alone and in combination) promoted differentiation through ATII-like cells, the highest percentage of surfactant protein C (SP-C) expressing cells (~37%) were produced in DE-like cells treated by F+H+CM. Ultrastructural analyses also confirmed the presence of lamellar bodies (LB) in the ATII-like cells.

**Conclusion:**

These results suggest that hydrocortisone can be a promoting factor in alveolar fate differentiation of IDE2- induced mESC-DE cells. These cells have potential for drug screening and cell-replacement therapies.

## Introduction

Pluripotent stem cells (PSCs) derived from lung epithelial cells can potentially be used in repair and regeneration of injured lung tissue and to model different respiratory diseases (1, 2). Among different lung epithelial cell types, alveolar epithelial type I/ alveolar epithelial type II (ATI/ATII) pneumocytes are of great interest. The main functions of ATII cells include the synthesis and secretion of surfactants (3), hyperplasia in reaction to alveolar epithelial injury (4), and renewal of the alveolar epithelium by acting as progenitor cells for ATI cells (5).

Mimicking lung developmental stages [reviewed in (6)] is an approach used to produce ATI/II cells from PSC sources *in vitro*. Several protocols have been published for definitive endoderm (DE) induction of PSCs (7). Studies reported the importance of transforming growth factor beta (TGF-ß) and Wnt3a signaling pathways for DE differentiation of PSCs. Theoretically, these PSC derived DE (PSC-DE) cells should be competent to further differentiate into endodermal derived cell types such as hepatocytes, pancreatic and lung cells. However, several studies have shown that PSC-DE cells exhibit different efficiencies when differentiated into these endodermal cell types (8, 9). Introduction of small-molecules to the differentiation protocols was a step towards more defined, xeno-free, universal methods for PSC differentiation [reviewed in (10)]. During high throughput chemical screenings, two small molecules [inducer of definitive endoderm 1/2 (IDE1/2)] were differentiation of murine and iPSCs, most probably by inducing the TGF-ß signaling pathway (11). 

A number of approaches have been reported to promote lung epithelial differentiation in PSC-DE cells. Primary studies used human lung carcinoma cell lines such as A549 or their conditioned medium (A549 CM) to induce lung differentiation (12). Different inducers from the fibroblast growth factor (FGF) family have been reported to be effective throughout the epithelialmesenchymal interaction during lung development (13). Beside growth factors, few small molecules such as dexamethasone (a glucocorticosteroid) have been reported which promote lung cell maturation and have been used in differentiation protocols (5, 14, 15). Hydrocortisone (or cortisol) is the most well-known glucocorticoid in the human body which is secreted by the adrenal gland. It induces both morphological and enzymatic changes in different tissues (16). While there is no report of the use of hydrocortisone for lung differentiation of PSCs, studies suggest important roles for this chemical in lung development, differentiation of ATII, and preterm manifestation of pulmonary surfactant (16, 17).

Hydrocortisone induces structural maturation of the embryonic lung and causes alveolar wall thinning, increases alveolar volume, and causes marked increase in pulmonary complications (17). In this study, we have tested the competency of IDE2-induced mouse embryonic stem cell (mESC) derived DE cells (mESC- DE) for alveolar differentiation by using different combinations of A549 CM, hydrocortisone, and FGF2.

## Materials and Methods

### Maintenance of mouse embryonic stem cells

The mESC line Royan B20 (RB20, passages 11-17, Royan Institute) were maintained in an undifferentiated state under feeder-free and serum-free R2i culture conditions as previously published (18) and described in the supplemental information section.

### Preparation of A549 conditioned medium 

Initially, the A549 cells were maintained in medium that contained Dulbecco’s minimum essential medium (DMEM, high glucose) and fetal bovine serum (FBS, all from Invitrogen, USA) in 5% CO_2_ at 95% humidity for 24 hours. Then, we changed the medium to DMEM (high glucose) and knockout serum replacement (KoSR, Invitrogen, USA) for 48 hours. Next, we collected and filtered the cell supernatant. The filtered supernatant was used in the differentiation protocol as A549 CM.

### Differentiation of mouse embryonic stem cells into definitive endoderm and alveolar epithelial type II cells

RB20 mESCs were induced to differentiate into DE (6 days) and then into ATII cells (9 days) by using the Mokhber Dezfouli et al. adherent culture method. During both steps, cultures were maintained in 5% CO2 and at 95% humidity with daily media changes.

### Differentiation protocol to definitive endoderm

The cells were washed with Dulbecco’s phosphate buffered saline (DPBS, Invitrogen, USA) prior to the addition of differentiation medium. To induce differentiation, mESCs were treated for 6 days in serum- free differentiation (SFD) media that consisted of DMEM/ F12 supplemented with N2, B27, 0.05% bovine serum albumin (BSA), 1% nonessential amino acids (NEAA), 1% L-glutamine, and 1% penicillin/streptomycin (all from Invitrogen, USA), and induced with 200 nM small molecule IDE2 (Stemgent, USA) (9).

### Differentiation into alveolar epithelial type II cells

For days 7-15 (9 days) of induction, we treated the resultant DE cells with new differentiation media comprised of 100 ng/ml basic FGF (bFGF) or FGF2 (Royan Institute, Iran), 0.5 µg/ml hydrocortisone (Invitrogen, USA), and A549 CM (filtered and added at a 50:50 v/v to the serum-free medium as the working solution) in SFD media. The experimental groups were divided into seven combinations of inductive factors, according to whether they received one, two or three inductive factors. Groups either received only one factor: FGF2 (group F), hydrocortisone (group H) or A549 CM (group CM); two factors: F+H, F+CM or H+CM; or all three inducers: F+H+CM. Therefore, the seven different combinations were used to test the efficiency of all contributions by these factors to promote differentiation.

### Isolation of RNA and real-time reverse transcriptase polymerase chain reaction

Isolation of total RNA and real-time reverse transcriptase polymerase chain reaction (RT-PCR) were performed as explained in supplemental information. Briefly, mESCs (day 0), DE (day 6) and ATII (day 15) cell cultures were collected. In addition, we obtained lung tissue from 30-day-old mice, washed the tissues three times with DPBS, and minced them into very small pieces. Total RNA was extracted by TRIzol (Invitrogen, USA). Contaminating DNA was removed with DNase I kit (Fermentas, USA), whereas RNA was guarded with RiboLock™ RNase inhibitor (Fermentas, USA). Total RNA was reverse transcribed by the RevertAid H Minus First Strand cDNA Synthesis Kit (Fermentas, USA). We analyzed expressions of the following genes in the different experimental groups: *POU5F1 (Oct-4), *Sox17*, Foxa2, Nkx2.1, SP-A, SP-B,* and surfactant protein c (*SP-C*). The comparative Ct, 2^-ΔΔCt^ method was used for relative gene expression analysis (19). The primers used were designed by Perl Primer software (20). Table S1 lists the primer sequences and the expected product sizes (See Supplementary Online Information at www.celljournal. org).

### Immunofluorescent staining

mESCs (day 0), DEFGf (day 6), and ATII (day 15) cell cultures were fixed with 4% paraformaldehyde (Sigma- Aldrich, USA) and permeabilized in 0.1% Triton X-100 (Sigma-Aldrich, USA). Cells were blocked in 10% antibody of the secondary host serum and subsequently incubated with the primary antibodies listed in Supplementary Table 2. Next, the cultures were incubated with the secondary antibodies (Table S2) (See Supplementary Online Information at www.celljournal.org). We used 4’,6-diamidino-2-phenylindole (DAPI, Sigma- Aldrich, USA) to stain the nuclei. Details of the methods are available in supplemental information.

### Flow cytometry analysis

mESCs (day 0), DE (day 6), and ATII (day 15) cell cultures were separated into single cell suspensions after incubation with 0.25% trypsin/EDTA, and then collected by centrifugation. The dissociated cells were resuspended in fixation/permeabilization solution. After blocking in 10% goat serum, the cells were incubated with the primary antibodies followed by incubation with the secondary antibodies (Table S2) (See Supplementary Online Information at www.celljournal.org). Detailed methods are available in the supplemental information section.

### Transmission electron microscopy analysis 

We processed the day 15 samples (ATII cells) for transmission electron microscopy (TEM) as previously described (21). Briefly, the samples were fixed using 2.5% glutaraldehyde in 0.1 M PBS (pH=7.4) for 2 hours at room temperature. After washing with DPBS, samples were post-fixed with 1% osmium tetroxide for 1.5 hours at room temperature, washed in DPBS, and progressively dehydrated in an acetone series. The resultant samples were subsequently embedded in epoxy resin. After polymerization with resin, approximately 50 nm sections were rifted and stained twice with uranyl acetate and lead citrate. Images were acquired by a Zeiss EM 900 TEM (Zeiss, Germany).

### Statistical analysis

We conducted all experiments with at least three independent biological repeats. Data from flow cytometry and real-time RT-PCR are shown as mean ± SD and tested for normality analysis of the parameters. The mean value differences were statistically evaluated with SPSS software (SPSS Inc., USA, version 24) using one-way analysis of variance (ANOVA), followed by Tukey’s test. P<0.05 were considered to be statistically significant.

## Results

### Production of definitive endoderm-like cells using small molecule inducer of definitive endoderm 2 

**Fig.1:**
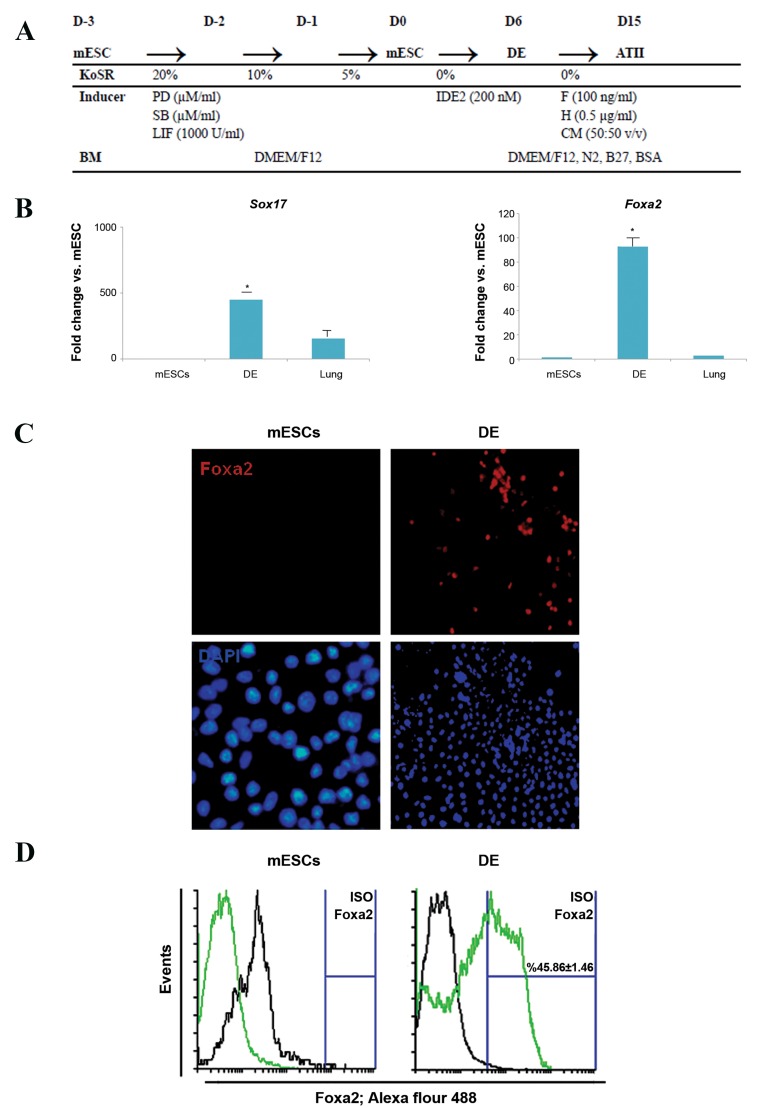
Small-molecule induced differentiation of mESCs towards DE-like cells. A. An overview of the mESC maintenance and differentiation protocol. Differentiationof the cells was initiated via a reduction in concentration of KoSR. After 3 days, cells were induced by IDE2 for 6 days. In the next step, DE cells differentiatedinto ATII-like cells by using 3 inductive factors: FGF2 (F), hydrocortisone (H), and A549 CM during 9 days, B. The expression levels of *Sox17* and *Foxa2* by RT-PCR increased significantly (*; P<0.05) by day 6 compared to mESCs, C. mESC-derived DE cells were immunostained by rabbit anti-goat *Foxa2* antibody (red) and nucleicounterstained with DAPI (blue). Lack of expression of *Foxa2* in mESC cells (scale bar: 100 µm), and D. Flow cytometry analysis showed increased numbers of cells that expressed the DE-specific marker, *Foxa2*, at the protein level. mESC; Mouse embryonic stem cell, DE; Definitive endoderm, KoSR; Knockout serum replacement, IDE2; Inducer of definitive endoderm 2, FGF; Fibroblast growthfactor, ATII-like; Alveolar epithelial type II-like cells, A549 CM; A549 conditioned medium, and RT-PCR; Real-time polymerase chain reaction.

RB20 mESCs were maintained in adherent culture conditions on gelatin-coated dishes prior to induction of differentiation. mESCs were cultured in media with reducing concentrations of serum (20, 10, and 5% FBS) over a 3-day period, followed by induction with 200 nM IDE2 for 6 days. At the end of stage 1, DE- like cells were characterized for the expression of two markers of DE-*Sox17* and *Foxa2*. Their morphology was assayed by phase contrast microscopy (Fig .1A).

At day 6 of IDE2 induction, cells showed the epithelial morphology characteristic of DE (Fig .S1) (See Supplementary Online Information at www. celljournal.org). Real-time RT-PCR results indicated increased expressions of *Sox17* and *Foxa2* by day 6 compared to the negative control group (Fig .1B). Immune staining and flow cytometry analysis also showed an increase in Foxa2 at the protein level (Fig .1C, D). 

### Induction of mouse embryonic stem cell-derived definitive endoderm towards alveolar epithelial type II-like cells using hydrocortisone containing medium

After 6 days induction with IDE2, DE-like cells were induced with 7 different differentiation media (Fig .1A). After 9 days, we analyzed the resultant cell population for different ATII-specific markers by gene and protein expression analyses. In all cases, we compared the results to DE-like cells (day 6) and mESCs (day 0). The resultant cells underwent morphological investigation by phase contrast microscopy and ultrastructural analysis by electron microscopy.

### Gene expression profile of differentiated alveolar epithelial type II-like cells

**Fig.2:**
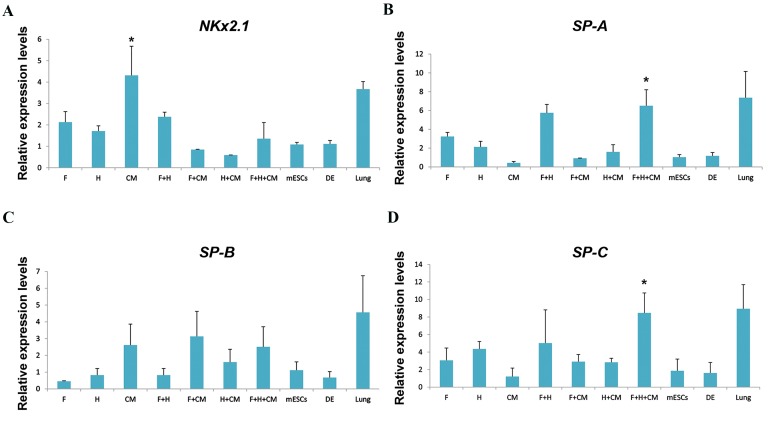
RT-PCR analysis of gene expression levels during differentiation into ATII cells. A-D. Expression levels of lung alveolar specific marker genes were analyzed in different experimental groups. The target gene expression level was normalized to GAPDH and presented relative to mESCs. Data are presented as mean ± SD. *; Significant to mESCs and DE groups, but not significant with positive control (lung) group. At least P<0.05 as determined by ANOVA with Tukey’s HSD test, n=3. RT-PCR; Reverse transcriptase polymerase chain reaction, FGF; Fibroblast growth factor, F; FGF2, H; Hydrocortisone, CM; A549 conditioned medium, mESC; Mouse embryonic stem cells as the negative control, DE; Definitive endoderm-like cells, and ATII; Alveolar epithelial type II cells.

The gene expression levels of pluripotent marker *Oct4*, DE-specific markers *Sox17* and *Foxa2* (Fig .S2) (See Supplementary Online Information at www. celljournal.org), and several important early and late ATII-specific genes (*Nkx2.1, SP-A, SP-B* and *SP-C*) were analyzed at days 0, 6 and 15 of differentiation (Fig .2). We conducted the gene expression experiments with undifferentiated ESCs as the negative control and lung tissue as the positive control. The results showed downregulation of *Oct4*, as a pluripotent marker, in all experimental groups compared with mESCs (Fig.S2A) (See Supplementary Online Information at www. celljournal.org). DE-specific markers (*Sox17* and *Foxa2*) significantly upregulated in the DE stage and subsequently downregulated after further induction towards ATII cells with different media (Fig .S2B, C) (See Supplementary Online Information at www.celljournal.org). Gene expression analysis at day 15 (ATII-like cells) showed significant upregulation of *SP-A* and *SP-C* (ATII-specific markers) in the F+H+CM group. Nkx2.1, the expressed marker in proximal and distal lung epithelial progenitors, upregulated in CM (Fig .2A-D).

### Surfactant protein C expression level in differentiated alveolar epithelial type II-like cells 

**Fig.3:**
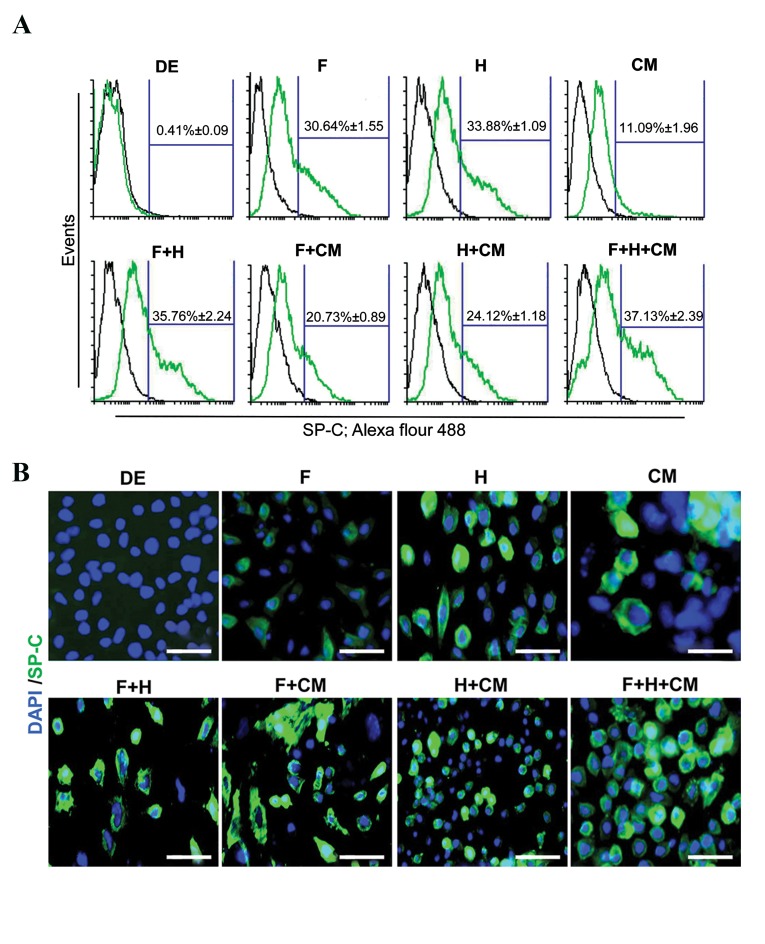
Flow cytometric analysis and immunofluorescent staining for SP-C as a unique marker of ATII cells. A. The numbers of SP-C positive cells were investigated in different stages of differentiation (mESCs, DE, and ATII) and different experimental groups. All F and H groups showed increased numbers of SP-C positive cells. The highest positive number of SP-C cells belonged to the F+H+CM group. Data are presented as mean ± SD and B. Cells in different stages of differentiation (mESCs, DE, and ATII) and different experimental groups immunostained by rabbit anti-goat SP-C antibody (green) and counterstained with DAPI (blue). The results agreed with the results of flow cytometry with the flow cytometry results (scale bar: 100 µm). FGF; Fibroblast growth factor, F; FGF2, H; Hydrocortisone, CM; A549 conditioned medium, mESC; Mouse embryonic stem cells as negative control, DE; Definitive endoderm-like cells, ATII; Lung alveolar type II-like cells, SP-C: Surfactant protein C.

SP-C, a unique marker of ATII cells, is commonly used to identify these cells from other lung parenchymal cell types (22). Flow cytometry (Fig .3A) and immunostaining (Fig .3B) analyses were performed to determine the level of SP-C in different experimental groups. The SP-C+cells were hardly detectable in day 0 mESCs (0.44 ± 0.07%, data not shown) and day 6 DE-like cells (0.41 ± 0.09%). However other differentiation protocols had detectable levels of SP-C+cells. Flow cytometry analysis indicated the highest number of SP-C+cells (37.13 ± 2.39%) in the F+H+CM group compared to the other groups (Fig .3A). 

**Fig.4:**
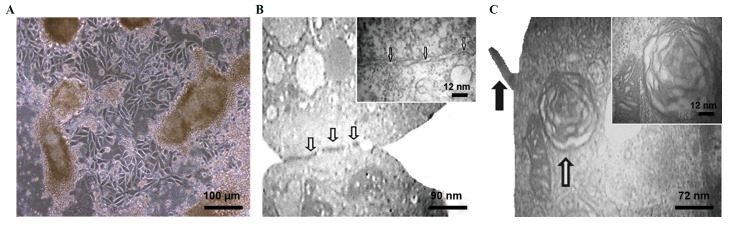
Ultrastructure of mESC-derived ATII-like cells. mESCs induced for 15 days in FGF2, hydrocortisone, and A549 conditioned medium (F+H+CM) were analyzedby phase contrast microscopy and TEM. A. Morphology of ATII-like cells at day 15 of culture in the F+H+CM group, B. The epithelial morphology of day 15 ATII-likecells showed lateral cell-cell contacts, which included tight junctions (arrows). Higher magnification also showed these structures, and C. Ultrastructure of day 15ATII-like cell shows microvilli and lamellar body (LB, black and white arrows, respectively). Higher magnification shows a well-developed LB with electron denselamellae in a multi-vesicular body. mESC; Mouse embryonic stem cell, ATII; Alveolar epithelial type II, FGF; Fibroblast growth factor, and TEM; Transmission electron microscopy.

### Ultra morphology of mouse embryonic stem cell- derived alveolar epithelial type II-like cells: presence of lamellar bodies

Cells induced by F+H+CM for 15 days were characterized morphologically by phase contrast microscopy (Fig .4A) and TEM (Fig .4B, C) in order to confirm the production of ATII-like cells at the ultrastructural level. The mESC-ATII cells exhibited ultrastructural features characteristic of mouse type II cells, which included apical microvilli (Fig .4B) and cytoplasmic LB (Fig .4C) as seen in the A549 cells (23).

## Discussion

Here, we described a two-step differentiation protocol. In the first step, mESCs were induced toward DE using IDE2. In the second step, the DE-like cells were differentiated into ATII cells by different inductive protocols. Our results showed that both F and H promoted ATII-like cell differentiation. The highest percentage of cells that expressed SP-C (~37%) were produced when DE-like cells were treated with F+H+CM. Ultrastructural analyses also confirmed the presence of lamellar bodies (LB) in ATII-like cells.

The introduction of novel small molecules is a step towards a better defined scalable protocol for DE production of ESCs. Several studies have reported small molecules that promote DE differentiation in ESCs (11, 24, 25). After a high throughput study, Borowiak et al. (11) introduced small molecules IDE1/2 as inducers of DE in both mouse and human ESCs. In agreement with this report, we found that DE-specific markers upregulated in mESCs after treatment of the cells with 200 nM IDE2. However, we could not induce DE differentiation in a human ESC line when these cells were treated with IDE2 during our previous studies (9, 26). While the study showed the competency of the IDE1/2-induced DE cells to differentiate into pancreatic progenitor cells (11), here we showed that DE-like cells had the capability to differentiate towards a lung epithelial fate by using the proper inductive conditions.

To promote differentiation toward ATII cells, we selected three previously introduced key factors (A549 CM, FGF2, and hydrocortisone). This research compared the ATII-inductive efficiency of these factors alone and in combination. Several studies used A549 CM, an ATII cell line derived from human lung carcinoma, to induce alveolar differentiation in ESC cells (27, 28). Although Roszell et al. (12) reported the inductive capacity of A549 CM for alveolar differentiation of mESCs, we found that A549 CM promoted upregulation of lung progenitor markers such as Nkx2.1 (29) and did not upregulate late markers of alveolar epithelium such as SP-C in the inducted DE cells. This observation agreed with a report which showed a heterogeneous population in mESCs treated with A549 CM (30). This finding could be attributed to the presence of growth factors in CM of A549 cells. The main growth factor families reported to promote differentiation into lung epithelial fate include FGF, bone morphogenic protein (BMP), and wingless-type mouse mammary tumor virus (Wnt) (12, 15, 31). Among these factors, different studies emphasized the importance of FGF signaling in embryonic lung development and morphogenesis (32, 33). Therefore, FGFs have been one of the main factors in ESC lung differentiation protocols (34). *In vivo* experiments conducted by Serls et al. (35) showed a dose-dependent fate determination of DE during development and suggested that the higher concentrations of this factor induced lung differentiation *in vivo*. This finding was confirmed *in vitro* when Roszell et al. (12) reported a higher number of mESC-ATII cells (~12.4% SP-C+cells) in cultures treated with a higher concentration (50 ng/ml vs. 5 ng/ml) of FGF2. Our results also confirmed that a high concentration of FGF2 (100 ng/ml) promoted ATII differentiation in mESC-DE cells (~30.6% of SP-C+ cells).

In the study, was introduced faster differentiation of human pluripotent stem cells (hPSCs) into functional airway epithelium by temporal regulation of canonical Wnt signaling via a progenitor of NKX2-1+progenitor cells (36). Dye et al. (37) reported differentiation of hPSCs into lung organoids. Initially they created anterior-ventral endoderm by modulation of FGF and SHH, and then developed pulmonary organoids that included pulmonary cells, smooth muscle, and myofibroblasts through NOG, FGF4, and CHIR99021.

*In vivo* studies indicated that in a normal fetus, an increase in circulating levels of endogenous corticosteroid could potentially increase the proportion of ATII and surfactant protein gene expressions (38, 39). While the impact of corticosteroids in lung developmental stages has been reported in pioneer literatures (17), few studies investigated the ability of these molecules to differentiate in PSC cultures. Schmeckebier et al. (5) introduced a 24day ATII differentiation protocol that used dexamethasone, as a synthetic corticosteroid, accompanied by FGF7. They observed upregulation of SP-C in mESCs. In our study, as a first report, we used hydrocortisone (a natural glucocorticoid) to induce differentiation of mESC-DE into ATII cells. Hydrocortisone has important roles in differentiation of ATII cells and the preterm manifestation of pulmonary surfactant (16, 17). We have observed approximately 30.6% SP-C+ cells in the DE cells during 9 days of treatment with hydrocortisone (group H). There are many similarities in structure and mechanisms of action between dexamethasone and hydrocortisone, however, differences exist in their potencies (40).

Phase contrast microscope examination demonstrated morphologically normal mESC-ATII cells. They consisted of cuboidal cells with a rounded core. TEM ultrastructural evaluation showed the presence of inclusion bodies (LB). LB is a hallmark used to identify ATII cells. LBs are intracellular structures that contain surfactant proteins and lipids (3). This finding has been confirmed by studies that reported generation of ATII cells *in vitro* (15, 22).

Our results indicated that groups F and H upregulated ATII- specific markers in treated DE cells. We observed the most significant upregulation in cells treated with the combination of F+H+CM. This result has provided further evidence that FGF2 and corticosteroids (hydrocortisone) are potent factors for differentiation of DE to lung ATII cells. 

## Conclusion

In the current study, we first showed that small molecule (IDE2)-induced DE cells had the capability to further differentiate into ATII cells. Secondly, we demonstrated the inductive capacity of hydrocortisone for ATII differentiation of DE cells. We have observed that hydrocortisone supported the generation of ATII cells from mESC-DE cells. These cells have potential for drug screening and cell-replacement therapies.

## Supplementary PDF


