# Supplementary material for: Hydrocortisone Promotes Differentiation of Mouse Embryonic Stem Cell-Derived Definitive Endoderm toward Lung Alveolar Epithelial Cells
Source: Cell J. 2018 Aug 7;20(4):469–76. doi: 10.22074/cellj.2019.5521 (PMC6099149; doi:10.22074/cellj.2019.5521)
Supplement: Supplementary file 1 [file Cell-J-20-469-s01.pdf]

CELL JOURNAL (Yakhteh)

www.celljournal.org

Supplementary Information for

Hydrocortisone Promotes Differentiation of Mouse Embryonic Stem Cell-Derived Definitive Endoderm toward Lung Alveolar Epithelial Cells

Mohammad Reza Mokhber Dezfouli, D.V.M., D.V.Sc.[1, 2]*, Sirous Sadeghian Chaleshtori, D.V.M., D.V.Sc.[1, 2], Azadeh Moradmand, M.Sc.[3], Mohsen Basiri, Ph.D.[3], Hossein Baharvand, Ph.D.[3, 4], Yaser Tahamtani, Ph.D.[3]*

1. Department of Internal Medicine, Faculty of Veterinary Medicine, University of Tehran, Tehran, Iran
2. Institute of Biomedical Research, Faculty of Veterinary Medicine, University of Tehran, Tehran, Iran
3. Department of Stem Cells and Developmental Biology, Cell Science Research Center, Royan Institute for Stem Cell Biology and Technology, ACECR, Tehran, Iran
4. Department of Developmental Biology, University of Science and Culture, ACECR, Tehran, Iran

**Corresponding Address: P.O.Box: 14155-6453, Department of Internal Medicine, Faculty of Veterinary Medicine, University of Tehran, Tehran, Iran*
P.O.Box: 16635-148, Department of Stem Cells and Developmental Biology, Cell Science Research Center, Royan Institute for Stem Cell Biology and Technology, ACECR, Tehran, Iran
Emails: mokhberd@ut.ac.ir, yasertahamtani@royaninstitute.org

Maintenance of mouse embryonic stem cell

Mouse embryonic stem cell (mESCs) line RB20 (passages 11-17, Royan Institute, Iran, Fig.S1A) were maintained in an undifferentiated state under feeder-free and serum-free R2i culture conditions as previously published (1). Briefly, the medium comprised DMEM/F12 (Invitrogen, USA), 20% KSR (Invitrogen), 1% NEAAs (Invitrogen), 1% L-glutamine (Invitrogen), 1% penicillin/streptomycin (Invitrogen), 0.1 mM β-mercaptoethanol (2ME, Sigma-Aldrich, USA), 1000 U mLIF (Chemicon, USA), 1 µM PD98059 (Stemgent, USA), and 10 µM SB431542 (Sigma-Aldrich). We changed the medium daily and cells were passaged in 0.1% gelatin-coated T-25 flasks (Falcon-Beckton Dickinson, San Jose, CA, USA) every 3-4 days with 1 ml trypsin/EDTA (Invitrogen) for 2-3 minutes, then resuspended in maintenance medium, and replated. Cultures were maintained in an undifferentiated state in 5% CO_2 and 95% humidity; the medium was changed daily. mESCs then differentiated into DE (Fig.S1B) and ATII.

RNA isolation and real-time reverse transcriptase polymerase chain reaction

mESCs (day 0), DE (day 6) and ATII (day 15) cell cultures were collected. Total RNA was extracted using TRIzol reagent (Invitrogen). DNA was degraded with the use of the DNase I, RNase-free kit (Fermentas, USA), whereas RNA was protected with RiboLock™ RNase Inhibitor (Fermentas). RNA was isolated from lung tissue that had been removed from 30-day-old mice, washed three times with DPBS, and cut into very small pieces. Total RNA was isolated using TRIzol reagent as previously described. RNA concentration was measured with a spectrophotometer (260/280 ratio: 1.8 to 2.0). RNA quality was verified using an electrophoresis system. Total RNA was reverse transcribed by the RevertAid H Minus First Strand cDNA Synthesis Kit (Fermentas) using a 0.2 µg random hexamer primer and 1 µg total RNA according to the manufacturer's instructions. Real-time reverse transcriptase polymerase chain reaction (RT-PCR) was performed in a Rotor-Gene 6000 instrument (Corbett Life Science, USA) with the following program: stage 1: 95ºC for 10 minutes, followed by 40 cycles of stage 2: 95ºC for 10 seconds, 60ºC for 30 seconds and 72ºC for 30 seconds. The PCR mix in each well included 10 µl of SYBR®Premix ExTaq™ II (RR081Q, Takara Bio, Inc.), 6 µl dH_2O, 1 ml each of the forward and reverse primers (5 pmol/µl), 2 µl of single strand cDNA (16 ng/µl) in a final reaction volume of 20 µl. We analyzed expressions of the following genes in the different experimental groups: *POU5F1* (*Oct-4*), *Sox17*, *Foxa2*, (Fig.S2) *Nkx2.1*, *SP-A*, *SP-B*, *SP-C* and *SP-D*. The comparative Ct method, $2^{-\Delta\Delta Ct}$, was used for relative gene expression analysis (2). The output data from Rotor-Gene 6000 analysis software (version 1.7, Corbett Life Science, USA) were transferred to Microsoft Excel for further analysis. For each sample, we calculated its relative expression level by normalization of the target genes to *Gapdh* as a reference gene, which was then calibrated against day 0 mESCs. In each experiment, we analyzed at least three independent replicates from each stage. Each replicate included two

identical samples. The primers used were designed with Perl Primer software (3). Primer sequences and expected product size are listed in Table S1.

Immunofluorescent staining

mESCs (day 0), DE (day 6) and ATII (day 15) cell cultures were washed with DPBS that contained 0.05% Tween-20 (PBST, pH=7.4, Sigma-Aldrich) and fixed with 4% paraformaldehyde (Sigma-Aldrich) in DPBS for 20 minutes at 4˚C. The cells were permeabilized in 0.1% Triton X-100 (Sigma-Aldrich) for 10 minutes at room temperature. Cells were twice washed and blocked in 10% secondary antibody host serum in washing buffer for 45 minutes at room temperature. The cell cultures were then incubated with primary antibodies that were diluted in washing buffer for 2 hours at 4˚C. Cells were subsequently washed three times with PBST (5 minutes each). Next, the cultures were incubated with the secondary antibodies (Table S2) at room temperature for 1 h and then washed three times with PBST (5 minutes each). Finally, 6-diamidino-2-phenylindole (DAPI, Sigma-Aldrich), a fluorescent dye, was used for staining nuclei for 5 minutes at room temperature. For negative controls, we omitted the primary antibodies and followed the same staining procedure (results not shown). We performed the experiment on at least three independent cell cultures for each antibody. Cells were observed under a fluorescence microscope (Olympus, BX51) and imaged with an Olympus DP72 digital camera mounted to the microscope.

Table S1: Real time reverse transcriptase polymerase chain reaction (RT-PCR) primers

Gene symbol	Sequences (5´- 3´)	Length (bp)
Sftpa (SP-A)	F: GCAAACAATGGGAGTCCTC<br>R: CGGCTCTGGTACACATCTCTC	119
Sftpb (SP-B)	F: GCTAGACAGGCAAAAGTGTGAAC<br>R: GGTGCAGGCTGAGGCTTGTC	210
Sftpc (SP-C)	F: ACCCTGTGTGGAGAGCTACCA<br>R: TTTGCGGAGGGTCTTTCCT	88
Sftpd (SP-D)	F: AGACAGAGGAATCAAAGGTG<br>R: AGGGAACAATGCAGCTTTCTGA	133
Nkx2.1	F: TTCCCCGCCATCTCCCGCT<br>R: TGTTCTTGCTCACGTCCCCCA	94
Foxa2	F: CGAGTTAAAGTATGCTGGGAG<br>R: CTATGTGTTCATGCCCATTCATC	123
Sox17	F : GATGTAAAGGTGAAAGGCGA<br>R : AAGACTTGCCTAGCATCTTG	196
Oct-4	F: GAA CTA GCA TTG AGA ACC GT<br>R: CAT ACT CGA ACC ACA TCC TTC	129
Gapdh	F: CAACTCCCACTCTTCCACTT<br>R: GCAGCGAACTTTATTGATGGGA	125

Table S2: Primary and secondary antibodies used for immunofluorescent staining and flow cytometry analyses

Antibody name	Host species	Manufacturer	Catalog number	Dilution factor
Primary antibodies				
Oct-4	Rabbit	Sigma Aldrich	AB3209	1:100
Foxa2	Rabbit	Sigma Aldrich	AB4125	1:100
Surfactant protein-C (SP-C)	Rabbit	Chemicon	ABC99	1:500
Secondary antibodies				
Goat IgG-alexa fluor 488	Rabbit	Invitrogen	A-11008	1:1000
Goat IgG-alexa fluor 594	Rabbit	Invitrogen	R37117	1:1000

Flow cytometry analysis

mESCs (day 0), DE (day 6), and ATII (day 15) cell cultures were dissociated into single cell suspensions by incubation with 0.25% trypsin/EDTA for 2 minutes at 37˚C, washed with DPBS, and collected by centrifugation. The dissociated cells were resuspended in fixation/permeabilization solution, kept at room temperature for 20 minutes, and washed twice with PBST. After blocking with 10% goat serum in washing buffer for 30 minutes at 37˚C, the cells were incubated with the primary antibodies for 2 hours at 4˚C. The cells were resuspended in 1 ml of PBST for 5 minutes followed by incubation with the secondary antibodies (Table S2) for 1 hour at 4˚C. Finally, the cells were washed twice with PBST and analyzed on a BD FACS Calibur flow cytometer (BD Biosciences). All experiments were performed in triplicate and the acquired data analyzed with Flowing Software (version 2.4, Turku Centre for Biotechnology).

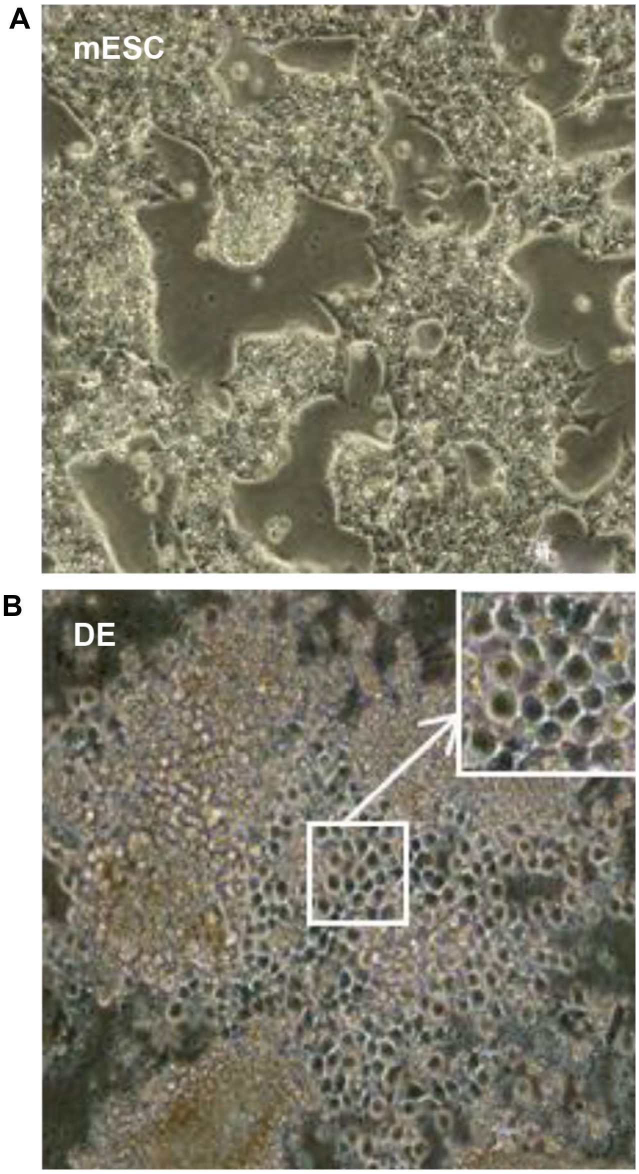

Fig.S1: Phase contrast micrograph of mouse embryonic stem cells (mESCs) versus definitive endoderm-like cells. **A.** Undifferentiated mESCs, and **B.** DE-like cells on day 6 of differentiation which showed an epithelial morphology cells (scale bar: 100 µm).

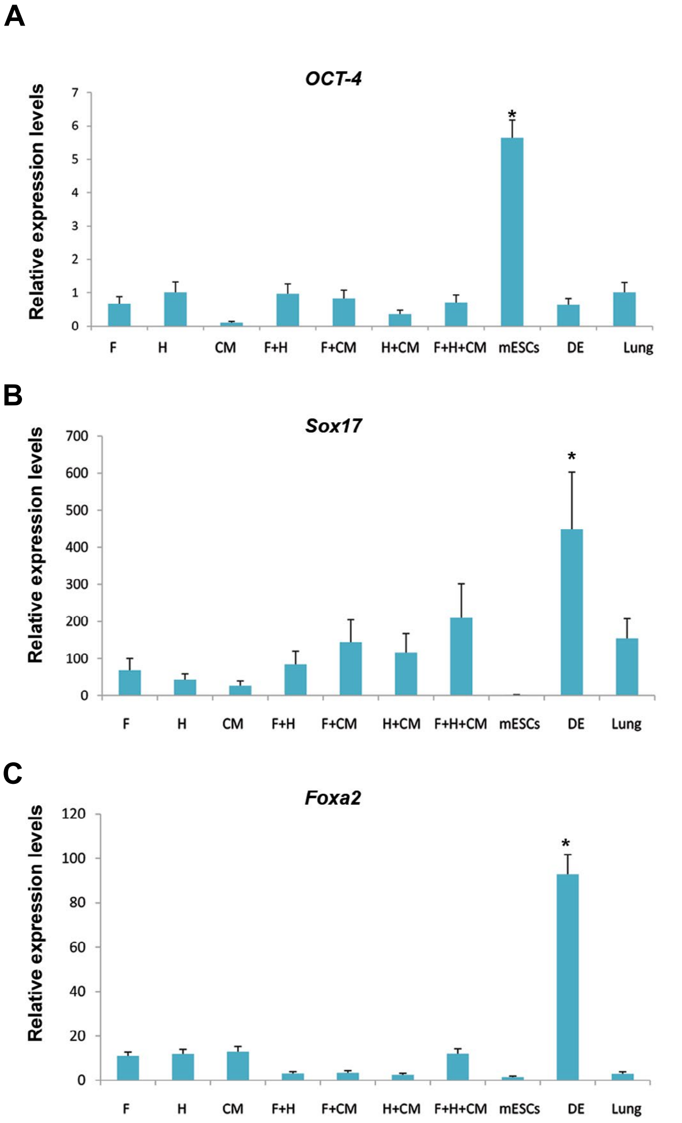

Fig.S2: Gene expression analysis during differentiation into DE by quantitative RT-PCR. Expression levels of **A.** Pluripotency and **B, C.** DE specific marker genes were analyzed during different stages of differentiation (mESCs and DE) and in different experimental groups. The target gene expression level was normalized to *GAPDH* and presented relative to mESCs. Data are presented as mean ± SD.
RT-PCR; Real-time reverse transcriptase polymerase chain reaction, FGF; Fibroblast growth factor, *; Significant to mESCs and DE groups, but not significant with positive control (lung) group. At least $P<0.05$ as determined by ANOVA with Tukey's HSD test, n=3, F; FGF2, H; Hydrocortisone, CM; A594 conditioned medium, mESC; Mouse embryonic stem cells as the negative control, DE; Definitive endoderm-like cells, and ATII; Lung alveolar type II-like cells.

References

1. Herriges JC, Verheyden JM, Zhang Z, Sui P, Zhang Y, Anderson MJ, et al. FGF-regulated ETV transcription factors control FGF-SHH feedback loop in lung branching. Dev Cell. 2015; 35(3): 322-332.
2. Sadeghian Chaleshtori S, Dezfouli MR, Dehghan MM, Tavanaei-manesh H. Generation of lung and airway epithelial cells from embryonic stem cells in vitro. Crit Rev Eukaryot Gene Expr. 2016; 26(1): 1-9.
3. Serls AE, Doherty S, Parvatiyar P, Wells JM, Deutsch GH. Different thresholds of fibroblast growth factors pattern the ventral foregut into liver and lung. Development. 2005; 132(1): 35-47.
